# Case Report: Anti-NMDAR Encephalitis Presenting With Catatonic Symptoms in an Adolescent Female Patient With a History of Traumatic Exposure

**DOI:** 10.3389/fpsyt.2022.784306

**Published:** 2022-01-28

**Authors:** Anamaria Bogdan, Florence Askenazy, Christian Richelme, Morgane Gindt, Susanne Thümmler, Arnaud Fernandez

**Affiliations:** ^1^Service Universitaire de Psychiatrie de l'Enfant et de l'Adolescent, Hôpitaux Pédiatriques de Nice CHU-Lenval, Nice, France; ^2^Université Côte d'Azur, CoBTek, FRIS, Nice, France; ^3^Service Universitaire de Pédiatrie, Hôpitaux Pédiatriques de Nice CHU-Lenval, Nice, France

**Keywords:** NMDAR encephalitis, catatonia, trauma, child and adolescent psychiatry, case report

## Abstract

**Introduction:**

Catatonia is a severe syndrome associated with a high proportion of underlying organic conditions including autoimmune encephalitis. The link between catatonia and psychiatric conditions such as mood disorders and schizophrenia spectrum disorders is well established while the causative effect of Post-Traumatic Stress Disorders and stress related disorders remains speculative.

**Case Report:**

Here we describe the clinical case of a 14-year-old female patient presenting to the Emergency Department of a Pediatric University Hospital with acute changes in behavior five days after a sexual abuse. Acute stress reaction was suspected. Afterwards she developed catatonic symptoms alternating from stupor to excitement, resistant to the usual treatment with benzodiazepines. The first line examinations (PE, MRI, EEG) were inconclusive. The final diagnosis of anti-NMDARE was made 22 days after her admission in a University Department of Child and Adolescent Psychiatry. Her state improved after first- and second-line immunotherapy, with no signs of relapse at this day (8 months of clinical follow-up).

**Discussion:**

The diagnosis of anti-NMDARE is challenging, involving a multidisciplinary approach. The neuropsychiatric features are complex, with no specific psychiatric phenotype. Several hypotheses are discussed to determine the role of an acute environmental stressors in the emergence of such complex neuropsychiatric clinical presentation (i.e., shared vulnerability, precipitators, consequences of preexisting psychiatric symptoms).

**Conclusion:**

Child and adolescent psychiatrists and pediatricians should be aware of the overlap between neurological and psychiatric features in the setting of anti-NMDARE. Catatonia should not be dismissed as a primary psychiatric disorder even in the context of recent traumatic exposure.

## Introduction

Catatonia is a rare but severe psychiatric syndrome, with a prevalence ranging from 0.6 to 17.7% in children and adolescents (1, 2), and it is associated with increased morbidity and a 60-fold increased risk of premature death including suicide (3). The estimated proportion of organic conditions (such as neurometabolic disorders, genetic conditions, autoimmune disorders) in pediatric catatonia is over 20 and 31% of patients present a history of developmental disorder (4). The link between catatonia and psychiatric conditions such as mood disorders and schizophrenia spectrum disorders is well established while the possible implications of Post-Traumatic Stress Disorder (PTSD) and stress related disorders remain speculative (2, 5). Anti NMDA-R encephalitis (anti-NMDARE), the most common form of autoimmune encephalitis in children (6, 7) is an autoimmune disorder mediated by autoantibodies against the GluN1 subunit of the receptor, presenting with prominent neuropsychiatric manifestations, including catatonia (8, 9). Eyre *et.al*., reported that 86% of children and adolescents with Anti-NMDARE had symptoms consistent with catatonia, with 69% of affected patients manifesting both positive (hyperkinetic) and negative (hypokinetic) features (10). About 80% of patients presenting with anti Anti-NMDARE are women and the detection of an underlying tumor varies with age, with younger people being less likely to have an underlying tumor (11). Symptom presentation varies between children and adults with children presenting more neurological features while adults and teenagers more psychiatric, leading to hospital admission for psychosis. In most cases the symptoms evolve toward a similar syndrome (6, 12). Psychotic symptoms resulting from Anti-NMDARE (delusions, hallucinations, mania, agitation, changes in speech, disorganized thinking, catatonia, insomnia) may not be distinct from those seen in schizophrenia and the glutamate hypothesis of schizophrenia emphasizes the central role of NMDAR in the pathophysiology of the disease (13). This explains why many patients are initially examined by a psychiatrist or admitted to psychiatric centers before being transferred to medical care units (6, 14). Furthermore, patients can present with isolated psychiatric symptoms (15), which may prompt to a diagnosis delay while early treatment with immunotherapy being associated with a better prognosis (6). Psychiatric presentations in patients with Anti-NMDARE are heterogenous with no specific anti-NMDARE psychiatric phenotype (9, 13, 16). The diagnosis of pediatric autoimmune encephalitis should be considered in patients presenting with focal diffuse neurologic deficits, movement abnormalities, autonomic instability, cognitive deficits, psychiatric symptoms and/or seizures with acute or subacute (less than 3 months) onset (17). This should prompt to a detailed workup, including brain MRI, EEG, and cerebrospinal fluid (CSF) analysis, to confirm the diagnosis and exclude other possibilities like infective encephalitis or systemic/metabolic causes (18). The definite diagnosis of anti-NMDARE is made in the presence of IgG anti-GluN1 antibodies, and antibody studies should include CSF analysis. Both CSF and serum should be used to reduce the risk of false-negative (seronegative encephalitis) or false-positive diagnosis. The concentration of CSF antibodies correlates better with the clinical course than antibody concentration in the serum (19). Treatment involves first-line immunotherapy (corticosteroids, intravenous immunoglobulins, or plasma exchange) and if needed, second line immunotherapy (rituximab, cyclophosphamide). For refractory patients, third-line treatments such as tocilizumab or bortezomib have been suggested (9). Patients presenting with anti-NMDARE should be screened for an associated neoplasm, and if a tumor is found (almost always an ovarian teratoma expressing NMDAR) the removal of the tumor is associated with an accelerated improvement and decreased relapse (6, 18, 20). The management of the psychiatric symptoms is challenging. Antipsychotic use should be considered carefully in this population, particularly when catatonia is present, neuroleptic sensitivity being an almost systematic feature of the disorder (13, 16).

Here we report the case of a 14-year-old female patient with an anti-NMDARE who initially presented to the emergency department after a sexual abuse.

## Case Report

The patient was brought to the Pediatric Emergency Department of a University Hospital by her mother for changes in behavior in the setting of a sexual abuse (during a party with friends and by a person invited but unknown to the patient) 5 days prior to the admission. The allegation was made at home, to her mother, prior to the emergency room visit. The recent history of the illness reveals an onset of symptoms 15 days prior to the sexual assault with irritability and a sad mood arguing for a mood disorder. After the sexual assault (which could also fit into this framework via behavioral disinhibition), the patient presented bizarre behavior (showering with clothes on, leaving the house without her shoes) that could suggest a psychotic disorder despite the absence of delusions and hallucinations. In the recent history of the disease, there was no evidence for a catatonic syndrome, the first symptoms (mutism) appeared on the day of the visit to the pediatric emergency room. The birth history is unremarkable, with no development delays, except learning difficulties since primary school due to dyslexia, with no other medical or psychiatric history. Family history is unremarkable for neurological, autoimmune, or psychiatric disorders, although her mother reported having an anxious temperament.

Upon her arrival the patient was conscious and afebrile. The neurological examination revealed no abnormalities. During the examination the patient was silent or responding briefly (mutism). Her blood count and usual biochemistry were normal with no signs of inflammation (CRP 0,5 mg/l), and the toxicological screen was negative. A diagnosis of acute stress reaction was suspected. Upon hospitalization in the pediatric ward, the patient became agitated, with incoherent speech (using words in an unappropriated manner). The initial EEG showed focal slow wave activity in the left anterior region and improved upon control.

The patient was transferred to the University Department of Child and Adolescent Psychiatry at day 1 where she developed symptoms consistent with a diagnosis of catatonia (excitement alternating with stupor, mutism, staring, grimacing, echolalia, stereotypy, verbigeration, negativism, refusal to eat and to drink and withdrawal). There was no autonomic instability except for a sinus tachycardia (140 bpm) that could be part of this malignant catatonia context but could also be related to dehydration or another medical condition. The Bush Francis Rating Scale (BFCRS) was 26. Diazepam IM was prescribed due to extreme agitation, with a partial response at one h (8 points improvement on the BFCRS), and Lorazepam *p.o* was continued at 10 mg per day. A diagnosis of non-infectious encephalitis was suspected, and a lumbar puncture (LP) was scheduled. On Day 2 to she developed vivid visual hallucinations (complex scenes involving people and animals trying to hurt her) and insomnia, with a persistence of the catatonic symptoms, alternation between extreme agitation and stupor. The cerebral MRI was normal.

On day 5, due to inadequate oral fluid intake, the patient became dehydrated with hypernatremia (Na 148 mmol/l). She was therefore retransferred to the pediatric ward to receive IV fluids. Due to dysphagia *p.o* treatment was stopped, and she was stared on clonazepam IV 4 mg/d. In addition to the worsening of catatonic symptoms (BFCRS 30 points) the patient developed seizures at day 10 requiring the introduction of antiepileptic treatment with Levetiracetam *p.o* 1000/day.

The LP was performed after somatic stabilization, showing moderate pleocytosis (*n* = 59 cells/mm3), with no signs of bacterial or viral infection (gram stain negative, sterile culture at day 3, multiplex PCR negative) and with a normal rate of proteins, glucose, and ions. A contrast cerebral MRI was ordered revealing a possible left frontal-insular hyperintensity with an uncertain signification due to dental material (metallic braces). The diagnosis of anti-NMDARE was confirmed by the presence of anti-NMDAR auto antibodies in the serum and in the CSF (Table 1 summarizes the diagnosis process in a chronological order).

**Table 1:** Summarizes the diagnostic process in a chronological order.

**Diagnostic process**		
Day 1	EEG	Slow left anterior wave. No paroxysmal anomaly.
Day 3	EEG	No signs of encephalopathy. Residual slow waves in anterior region. Improvement.
	MRI	Normal. Movement and dental material artifacts.
Day 8	EEG	Diffuse slowing. No paroxysmal anomaly.
Day 10	EEG	Post-critical epileptic activity due to encephalopathy.
	Contrast MRI under general anesthesia	Possible minimal left frontal-insular hyperintensities in FLAIR.
Day 11	LP	Moderate pleocytosis *n* = 59 cells/mm3.
Day 22		Confirmation of anti -NMDAR antibodies

IV corticosteroids (methylprednisolone 20 mg/kg/d) were started at day 11 and continued for 3 days. After the diagnosis was confirmed, five sessions of plasma exchange were performed over a 10-day period fallowed by IV Immunoglobulins (1 g/kg) over a 2-day period. The patient showed only small improvement, motivating the introduction of a second line therapy (Rituximab). The pelvic, abdominal, and thoracic scanner as well as the abdominal ultrasound were normal, excluding the presence of a macroscopic teratoma.

She was discharged to a rehabilitation pediatric clinic. There is no sign of relapse at this day, although she presents aggressive behavior, making the rehabilitation difficult. A treatment with low dose quetiapine was proposed.

## Discussion

Since the discovery in 2005, psychiatrists are increasingly involved in the diagnosis and management of patients presenting with anti-NMDARE (9, 10). The neuropsychiatric symptoms of Anti-NMDARE are complex. Patients can present with agitation, mood changes, psychosis. Movement disorders including catatonia are a common and complex feature of the disease (13, 21). Catatonic symptoms seem resistant to lorazepam, and this resistance should prompt for an evaluation of an underlying neurological disorder (10). Furthermore, catatonia is associated with more severe forms of autoimmune disorders and with more psychotic features (22). A symptomatic treatment with benzodiazepine should be started when a diagnosis of catatonia is established and continued until etiological treatment medication (immunothery) shows benefit. For residual catatonic symptoms despite immunosuppressive treatment, electroconvulsive therapy (ECT) might be considered (23). Catatonic symptoms associated with anti-NMDARE showed some improvement after ECT, with a favorable risk-to-benefit ratio (24), even in children (25). Although the mechanism of action of ECT remains unclear, it has been suggested that ECT may upregulate the NMDAR, thus being effective in the treatment of Anti-NMDARE (26). Thus, evidence suggest that ECT is broadly effective for pediatric catatonia, even when it is underpinned by Anti-NMDARE.

In our patient, organic catatonia was suspected since day 1, and the clinical course involving a rich psychiatric syndrome resistant to usual treatment, the development of epileptic seizures and the CSF pleocytosis increased the probability of autoimmune encephalitis even in the absence of clear MRI signs, diagnosis which was later confirmed by the anti-NMDAR antibody count.

The delay between the symptom-onset and the definitive diagnosis is explained not only by the diagnostic challenges involving a multidisciplinary approach, but also by logistical factors, the anti-NMDAR antibodies testing being done in a reference center in another city.

Graus et al., proposed diagnostic guidelines for autoimmune encephalitis mainly for the adult population, based on clinical levels of evidence, emphasizing the idea that the antibody testing should not be a part of the early diagnostic criteria and a diagnosis of autoimmune encephalitis should not be excluded by the absence of specific auto-antibodies (19). In the pediatric population a casualty assessment score (CAUS) was designed to discriminate organic catatonia (including autoimmune catatonia) from the non-organic form, with an excellent performance, in a cohort of 104 catatonic children and adolescents. The score is designed as a stepwise approach, involving large biological and paraclinical screening and an immunosuppressive therapeutic challenge (23), and represents a practical diagnosis tool for clinicians.

Our patient was wearing metal dental material, leading to artifacts on conventional imagery used in the evaluation of patients with catatonia. At this day, the proposed diagnostic framework for autoimmune encephalitis does not involve 8fluorodeoxyglucose (8F-FDG) PET imaging (27). 8F-FDG PET imaging seems to have higher sensitivity compared to conventional imagery in detecting autoimmune encephalitis (28) even in children, although no pathognomonic pattern for Anti-NMDARE emerged in this population (29). There are limits in the use of this technique in current practice. First of all, it is unavailable in many hospitals, and cannot be obtained on an emergency basis (30). Furthermore, there is reluctance in the usage of nuclear imaging in pediatric patients due to the perception of the radiation burden associated with this technique, so cases should be carefully selected (29). It has been suggested that 8F-FDG PET might have a diagnostic role in autoantibody-negative encephalitis and that it might play a role in monitoring of the immunosuppressive response (27, 30), even in pediatric population (31), but further studies are needed.

Antipsychotics should be used with caution in this population (13). Patients may develop hyperthermia, muscle rigidity, coma or rhabdomyolysis that suggest neuroleptic intolerance, but this symptoms may develop in neuroleptic-naive patients with anti-NMDARE making it difficult to distinguish between neuroleptic malignant syndrome and genuine symptoms of the disease (9). Due to its profile, with low D2 binding and high affinity for noradrenergic and histamine type I receptors, quetiapine has been successfully used in the treatment of agitation (common feature of anti-NMDARE) in pediatric patients, being less likely to cause extrapyramidal symptoms or neuroleptic malignant syndrome (32).

Tumors, mostly ovarian teratomas and herpes simplex encephalitis are two common triggers of anti-NMDARE, and two studies suggested the association of the B^*^07:02 HLA-I and DRB1^*^16:02 HLA-II alleles with a disease susceptibility, but underlying genetic mechanisms remain unclear (9). In our patient the tumor screening was negative and there was no sign of viral encephalitis (viral PCR negative in the CSF). She was the victim of sexual abuse which may prompt to acute stress symptoms and PTSD. In a recent study involving adult patients, a history of PTSD was associated with an increased risk of Systemic Lupus Erythematosus (SLE) (33). Furthermore, patients presenting with other stress-related disorders more common than PTSD, including acute stress reaction, present an increased risk of autoimmune disorders (34). This association may be mediated by inflammation, as shown in previous studies. Interleukin 1β, interleukin 6 and interferon γ levels are higher in patients with PTSD than in healthy controls (35). The seroprevalence of anti NMDAR auto-antibodies is increased in chronic stress. Environmental stress may have a role in the development of Anti-NMDARE via different mechanisms, including the increased permeability of the blood brain barrier, the formation of peripheral antibodies or the intrathecal memory B cells (36).

The following hypotheses were formulated to consider the link between a traumatic event (sexual abuse) and the development of an autoimmune pathology (Anti-NMDARE) in our patient:

- Behavioral disinhibition due to catatonic symptoms or associated psychiatric symptoms (*e.g*., a mixed/manic episode) increases the risk of sexual abuse (stress generation theory). However, our patient does not have a medical history of mood disorder, nor did she develop an authentic manic episode requiring stabilizers as for the case presented by Consoli et al. (37). Thus, our main hypothesis seems to be behavioral disinhibition due to catatonic symptoms.
- Adverse Childhood Experiences (ACE) increase the risk of metabolic disease and possibly autoimmune disease (allostatic load hypothesis). However, our patient did not present this type of event in her personal history. Furthermore, no strong correlation was found between ACE and pediatric catatonia in a recent study (38) and ACE cannot be extrapolated to acute stress because it is not the same clinical entity.
- The concept of catatonia was proposed as a fear response (a form of stress-related catatonic episode) linking it to the animal defense reactions (5, 39, 40).
- An infectious disease (intercurrent or sexually transmitted). However, the patient did not present clinico-biological arguments for an intercurrent infection (no fever, negative PCR) and was also negative for sexually transmitted infections three months after her sexual assault (HIV, hepatitis B and C, Chlamydia trachomatis, Gonococci and HSV).
- Purely incidental (Berkson's bias) (5, 39, 40).

## Conclusion

Child and adolescent psychiatrists and pediatricians should be aware of the overlap of neurological and psychiatric symptoms in the setting of anti-NMDARE. Catatonia should not be dismissed as a primary psychiatric disorder even in the context of a recent traumatic exposure with normal neurological examination or soft neurological signs and patients should be carefully assessed for an underlying organic condition. Catatonic symptoms should be systematically evaluated with validated specific scales (41).

To our knowledge, this is the first case of Anti-NMDARE revealed by a traumatic exposure described in the indexed literature. Further studies are needed to establish a putative effect of acute stress in the development of autoimmune encephalitis.

## Data Availability Statement

The original contributions presented in the study are included in the article/supplementary files, further inquiries can be directed to the corresponding author/s.

## Ethics Statement

Ethical review and approval was not required for the study on human participants in accordance with the local legislation and institutional requirements. Written informed consent to participate in this study was provided by the participants' legal guardian/next of kin.

## Author Contributions

AB, FA, ST, and AF wrote the first draft of the manuscript. CR wrote sections of the manuscript. AB, FA, ST, CR, and AF contributed to manuscript revision, read, and approved the submitted version. AF, AB, and MG contributed to manuscript revision including responses to reviewers. All authors approved the submitted revised version.

## Conflict of Interest

The authors declare that the research was conducted in the absence of any commercial or financial relationships that could be construed as a potential conflict of interest.

## Publisher's Note

All claims expressed in this article are solely those of the authors and do not necessarily represent those of their affiliated organizations, or those of the publisher, the editors and the reviewers. Any product that may be evaluated in this article, or claim that may be made by its manufacturer, is not guaranteed or endorsed by the publisher.
